# Early metabolic markers identify potential targets for the prevention of type 2 diabetes

**DOI:** 10.1007/s00125-017-4325-0

**Published:** 2017-06-08

**Authors:** Gopal Peddinti, Jeff Cobb, Loic Yengo, Philippe Froguel, Jasmina Kravić, Beverley Balkau, Tiinamaija Tuomi, Tero Aittokallio, Leif Groop

**Affiliations:** 1 Institute for Molecular Medicine Finland (FIMM), Nordic EMBL Partnership for Molecular Medicine, University of Helsinki, Helsinki, Finland; 2 Tietotie 2, P. O. Box 1000, FIN-02044 VTT Espoo, Finland; 3 Metabolon Inc., Durham, NC USA; 4 CNRS UMR8199, Pasteur Institute of Lille, Lille, France; 5 European Genomic Institute for Diabetes (EGID), FR-3508 Lille, France; 6 Lille University, Lille, France; 7 Institute for Molecular Bioscience, The University of Queensland, Brisbane, QLD Australia; 8 Department of Genomics of Common Disease, School of Public Health, Imperial College London, Hammersmith Hospital, London, UK; 9 Lund University Diabetes Center, Lund, Sweden; 10 CESP, Faculty of Medicine – University Paris-South; Faculty of Medicine – University Versailles-St Quentin; Inserm U1018, University Paris-Saclay, Villejuif, France; 11 Department of Endocrinology, Abdominal Centre, Helsinki University Central Hospital, Helsinki, Finland; 12 Folkhalsan Research Center and Research Programs Unit, Diabetes and Obesity, University of Helsinki, Helsinki, Finland; 13 Department of Mathematics and Statistics, University of Turku, Turku, Finland

**Keywords:** Biomarkers, Early prediction, Kallikrein–kinin system, Machine learning, Metabolomics, Multivariate models, Prevention, Risk classification

## Abstract

**Aims/hypothesis:**

The aims of this study were to evaluate systematically the predictive power of comprehensive metabolomics profiles in predicting the future risk of type 2 diabetes, and to identify a panel of the most predictive metabolic markers.

**Methods:**

We applied an unbiased systems medicine approach to mine metabolite combinations that provide added value in predicting the future incidence of type 2 diabetes beyond known risk factors. We performed mass spectrometry-based targeted, as well as global untargeted, metabolomics, measuring a total of 568 metabolites, in a Finnish cohort of 543 non-diabetic individuals from the Botnia Prospective Study, which included 146 individuals who progressed to type 2 diabetes by the end of a 10 year follow-up period. Multivariate logistic regression was used to assess statistical associations, and regularised least-squares modelling was used to perform machine learning-based risk classification and marker selection. The predictive performance of the machine learning models and marker panels was evaluated using repeated nested cross-validation, and replicated in an independent French cohort of 1044 individuals including 231 participants who progressed to type 2 diabetes during a 9 year follow-up period in the DESIR (Data from an Epidemiological Study on the Insulin Resistance Syndrome) study.

**Results:**

Nine metabolites were negatively associated (potentially protective) and 25 were positively associated with progression to type 2 diabetes. Machine learning models based on the entire metabolome predicted progression to type 2 diabetes (area under the receiver operating characteristic curve, AUC = 0.77) significantly better than the reference model based on clinical risk factors alone (AUC = 0.68; DeLong’s *p* = 0.0009). The panel of metabolic markers selected by the machine learning-based feature selection also significantly improved the predictive performance over the reference model (AUC = 0.78; *p* = 0.00019; integrated discrimination improvement, IDI = 66.7%). This approach identified novel predictive biomarkers, such as α-tocopherol, bradykinin hydroxyproline, X-12063 and X-13435, which showed added value in predicting progression to type 2 diabetes when combined with known biomarkers such as glucose, mannose and α-hydroxybutyrate and routinely used clinical risk factors.

**Conclusions/interpretation:**

This study provides a panel of novel metabolic markers for future efforts aimed at the prevention of type 2 diabetes.

**Electronic supplementary material:**

The online version of this article (doi:10.1007/s00125-017-4325-0) contains peer-reviewed but unedited supplementary material, which is available to authorised users.

## Introduction

Type 2 diabetes is a major disease that affects more than 420 million individuals worldwide; if current trends continue, the number will surpass 700 million individuals by 2025 [1]. Predictive biomarkers are needed to allow physicians to identify and monitor individuals at high risk for the disease. Metabolomic profiling of the complete set of small-molecule metabolites allows for capturing physiological and pathophysiological changes in the body [2, 3]. Metabolomics has proved to be a rich source of markers for diabetes [4–9], and improved the prediction of type 2 diabetes incidence beyond clinical and biological markers [10]. For instance, untargeted plasma metabolomics measuring 447 metabolites in a large cohort of women from the TwinsUK study found metabolites associated with hyperglycaemia and type 2 diabetes [11], revealing a large set of potential metabolic markers including amino acids, carbohydrates, lipids, xenobiotics and unknowns, and highlighted an important role for the catabolism of branched chain amino acids (BCAAs) in type 2 diabetes. Another untargeted metabolomics study measured more than 4500 metabolites in a prospective cohort of 300 individuals who developed type 2 diabetes during 6 years follow-up and 300 matched control participants, and identified several metabolic alterations in lipid metabolism and sugars [12]. A recent meta-analysis of 19 prospective and 27 cross-sectional studies revealed the association of several metabolites with the incidence of prediabetes (i.e. impaired glucose tolerance, impaired fasting glucose, insulin resistance or impaired insulin sensitivity) and type 2 diabetes, including hexoses, aromatic amino acids, phospholipids and triacylglycerols, and confirmed the key role of BCAAs and aromatic amino acids in the prediction of type 2 diabetes [9].

Wang et al measured 61 metabolites and examined whether multi-metabolite panels could jointly predict the type 2 diabetes risk in 2422 normoglycaemic individuals followed for 12 years in the Framingham Offspring Study [13]. They showed that BCAAs and aromatic amino acids were significantly associated with the future risk of type 2 diabetes, and that the combination of isoleucine, tyrosine and phenylalanine predicted the risk. Gall et al used untargeted metabolomics in a cohort of 399 non-diabetic individuals from the RISC (Relationship of Insulin Sensitivity to Cardiovascular Risk) study, and identified α-hydroxybutyrate (α-HB) as an early biomarker for insulin resistance and glucose intolerance [14]. Using the entire RISC cohort and a long-term observational cohort of at-risk individuals in the Botnia Prospective Study (BPS), Ferrannini et al performed targeted profiling of α-HB and 1-linoleoyl glycerophosphocholine (L-GPC) and showed that these markers jointly predicted insulin resistance and glucose intolerance [15]. These studies indicate that alterations in blood metabolite concentrations presage the onset of type 2 diabetes and aid in the identification of at-risk individuals by adding predictive information over standard clinical markers.

The objectives of the present data-driven study were to systematically assess the added value of comprehensive metabolomics data in predicting type 2 diabetes risk using machine learning-based predictive modelling, and to examine whether an unbiased feature-selection approach could identify novel metabolic marker combinations that improve the predictive performance over known biomarkers and clinical risk factors. We performed serum metabolomics in a prospective, follow-up study cohort of 543 non-diabetic individuals from the BPS, 146 of whom developed type 2 diabetes during a 10 year follow-up period. Predictive modelling enabled us to accurately predict the future incidence of type 2 diabetes using a novel biomarker panel.

## Methods

### Study population

The BPS was initiated in 1990 on the west coast of Finland to identify genetic factors contributing to type 2 diabetes, and includes a cohort of 2770 non-diabetic individuals followed for 10 years (median 7.7 years), 150 of whom developed type 2 diabetes [16]. A subpopulation of this cohort comprising 543 participants, whose fasting serum samples were available for metabolomics analyses, was used in the current study. This subpopulation thus included 146 participants who had progressed to type 2 diabetes by the end of the follow-up period, and 397 individuals randomly selected from those who did not progress to type 2 diabetes, such that sex is balanced. The ethics committee of the Helsinki University Hospital approved the study protocols. All individuals gave their informed consent to participation in the study.

Fasting serum samples collected at baseline were used for metabolomic profiling. Sex, age, BMI, fasting glucose, fasting insulin, family history of type 2 diabetes, waist circumference, systolic BP (SBP), diastolic BP (DBP), total blood cholesterol, HDL-cholesterol and triacylglycerols were recorded at baseline. The level of physical activity, use of antihypertensive medication (i.e. diuretics, beta blockers, calcium blockers, ACE inhibitors, angiotensin II receptor type 2 receptor inhibitors or other BP medication) and incidence of cardiovascular disease (CVD) during the follow-up period were also recorded. Glucose values measured using an OGTT at the end of the follow-up period were used to define whether an individual had progressed to type 2 diabetes (termed ‘progressors’) or remained non-diabetic (termed ‘non-progressors’).

For replication of our results, we used untargeted metabolomics data from fasting plasma samples of 1044 participants in the DESIR (Data from an Epidemiological Study on the Insulin Resistance Syndrome) study [10] from central western France, of whom 231 progressed to type 2 diabetes during a 9 year follow-up period (electronic supplementary material [ESM] Table 1). The ethics committee for the Protection of Subjects for Biomedical Research of Bicêtre Hospital, France, approved the study protocols. All individuals gave written informed consent to their participation in the study.

### Metabolomics

Samples were prepared using a single-extraction method. Global untargeted metabolomics was performed using three platforms (ultra-HPLC [UHPLC]-MS in electrospray ionisation-positive and -negative modes, and GC-MS in electrospray ionisation-positive mode) to semi-quantitatively measure a diverse set of 542 serum metabolites. Targeted metabolomics was performed using an isotope-dilution UHPLC-MS/MS assay for the absolute quantification of 26 metabolites. Metabolites were identified by automated spectral comparison with a standard library and missing values were imputed using minimum non-missing measurement [14, 17]. See ESM Methods for further details. The targeted and untargeted metabolomics data were further standardised to zero mean and unit variance per metabolite, and combined into a single data matrix containing 568 metabolite measurements from 543 samples. The DESIR validation data were similarly acquired [10].

### Statistical analysis

Fisher’s exact test was used to compare sex, family history, physical activity, use of antihypertensive medication and the presence of CVD between progressors and non-progressors, while Welch’s two-sample *t* test was used to compare age, BMI, fasting glucose, fasting insulin, waist size, SBP, DBP, cholesterol, HDL-cholesterol and triacylglycerols. Individuals with missing measurements for any of the clinical factors were excluded from all of the analyses involving that particular factor.

The statistical association of each metabolite with type 2 diabetes risk was assessed using logistic regression, using progression to type 2 diabetes as the binary response variable and the metabolite as the independent variable. ORs and corresponding significance levels (*p* value) were calculated from the logistic regression. *Q* values were calculated to control for the false discovery rate (FDR) [18]. Results at *q* < 0.05 were considered significant. For each significant metabolite, to assess whether its association with progression to type 2 diabetes was independent of the clinical risk factors, we performed multivariable logistic regression using the clinical covariates as additional variables.

### Predictive modelling

We used machine learning to build predictive models for future type 2 diabetes risk and to find the most predictive biomarker combinations. In contrast to univariate statistical analysis, predictive modelling uses the joint distribution of the metabolic features to build multivariate models, while employing model regularisation to prevent model overfitting and to enable generalisation to new individuals. Regularised least-squares (RLS) regression for binary risk classification was used to build the metabolome-wide predictive model. To select a minimal set of predictive metabolites, we used an efficient greedy feature-selection approach for RLS (GreedyRLS) [19].

To carefully assess the predictive performance of the RLS models beyond training data, we designed a repeated nested stratified cross-validation approach (ESM Fig. 1) [20]. Ten folds of outer cross-validation nested over ten folds of inner cross-validation were repeated 100 times, with stratified fold splitting to balance the numbers of progressors and non-progressors across the folds. The outer cross-validation estimated the prediction performance of the model, while the inner cross-validation selected the regularisation parameter and the linear coefficients. When applying GreedyRLS, we used the regularisation parameter selected in the outer cross-validation and the entire training data to determine the selected features. Repetition of the nested cross-validation ensured that the estimated prediction performance and the selected features were not due to any single random fold-split in the outer cross-validation. We reported the union of feature sets selected in 100 repetitions as the final biomarker panel.

Receiver operating characteristic (ROC) curves were also derived based on the repeated nested cross-validation. The mean of the AUC values was calculated from 100 ROC curves and the 95% CI for the AUC was calculated as 2.5th and 97.5th percentile values. DeLong’s test for correlated ROC curves was used to assess the pairwise differences between competing models [21]. The DESIR validation data were also predicted using model parameters obtained in 100 repeats and averaged to calculate the ROC curve. The 95% CI of the validation AUC was calculated using DeLong’s method [21]. R packages ROCR [22] and pROC [23] were used for ROC curve analyses.

We used integrated discrimination improvement (IDI) to evaluate whether metabolites improved type 2 diabetes risk prediction when combined with clinical risk factors [24]. To calculate IDI, the RLS-based risk scores were converted into risk probabilities by scaling with SD and applying logit transformation. The discrimination slope (DS) of a model provides a measure of its discriminative ability, similar to AUC. DS was calculated as the difference in the mean risk probability between progressors and non-progressors. IDI measures the improvement obtained by adding new predictors and was calculated as the difference in DS between models with and without the new predictors, and expressed as percentage improvement obtained in DS [24]. IDI is equivalent to the integration of the net reclassification improvement over all cut-offs for the risk probability.

## Results

We performed comprehensive metabolomics experiments and predictive modelling in 543 individuals from the BPS, including 146 progressors to type 2 diabetes during a 10 year follow-up period. The progressors and non-progressors were balanced for sex (Table 1). All individuals had normal glucose and insulin levels, but borderline-high cholesterol at the beginning of the study. Progressors were older and had higher BMI, fasting glucose and fasting insulin levels, waist size, SBP, DBP, and triacylglycerols than non-progressors, while HDL-cholesterol showed the opposite trend and total cholesterol showed no difference (Table 1).

**Table 1:** Clinical characteristics of individuals from the BPS used in this study, for training predictive models

Variable	Total population	Non-progressors	Progressors	*p* value
*n*	543	397	146	
Sex				
Male	274	200	74	1
Female	269	197	72	
Age (years)	49.33 ± 0.59	48.22 ± 0.72	52.34 ± 0.99	0.00089
BMI (kg/m^2^)	26.59 ± 0.18	25.91 ± 0.19	28.46 ± 0.37	4.5 × 10^−9^
Waist circumference (cm)	90.34 ± 0.54	88.19 ± 0.59	96.15 ± 1.04	2.2 × 10^−10^
Fasting glucose (mmol/l)	5.68 ± 0.03	5.60 ± 0.03	5.90 ± 0.05	8.0 × 10^−7^
Fasting insulin (pmol/l)	44.33 ± 1.61	38.85 ± 1.26	59.34 ± 4.71	4.4 × 10^−5^
SBP (mmHg)	132 ± 0.84	129.47 ± 0.95	138.87 ± 1.60	9.6 × 10^−7^
DBP (mmHg)	80.11 ± 0.47	78.70 ± 0.55	83.94 ± 0.86	5.2 × 10^−7^
Total cholesterol (mmol/l)	5.71 ± 0.05	5.68 ± 0.07	5.81 ± 0.09	0.21
HDL-cholesterol (mmol/l)	1.33 ± 0.01	1.35 ± 0.01	1.27 ± 0.03	0.02
Triacylglycerols (mmol/l)	1.44 ± 0.04	1.34 ± 0.04	1.69 ± 0.08	0.00014
Family history of type 2 diabetes				
No	30	27	3	0.01
Yes	289	190	99	
Data missing	224	180	44	
Future CVD				
No	419	305	114	1
Yes	124	92	32	
Physical activity				
Low	18	12	6	0.07
Medium	315	222	93	
High	184	146	38	
Data missing	26	17	9	
Hypertension medication				
No	275	214	61	1
Yes	60	41	19	
Data missing	208	142	66	

Data are *n* or means ± SEM.

Targeted metabolomics measured 26 metabolites and untargeted metabolomics detected 542 distinct metabolites (316 identified and 226 unidentified) in the serum samples. Metabolon (Durham, NC, USA) identifiers are used to refer to the unknown metabolites (e.g. X-13435).

### Individual metabolites are associated with type 2 diabetes risk

**Fig. 1:**
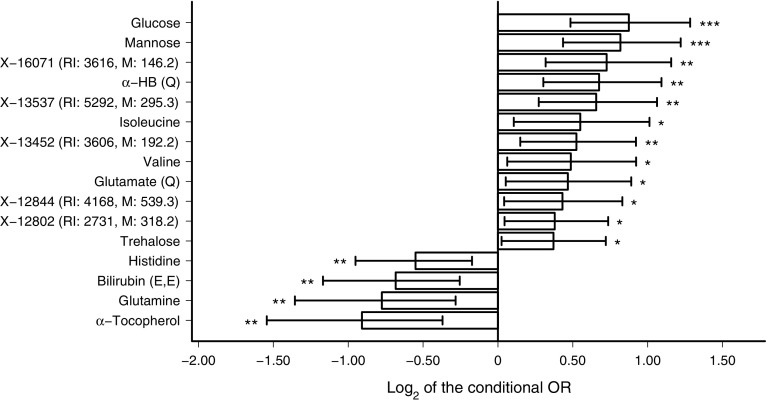
Metabolites associated with progression to type 2 diabetes at FDR *q* < 0.05. The figure shows conditional ORs, accounting for the risk factors age, sex, BMI, fasting insulin level and family history at baseline. Error bars indicate the 95% CI. Metabolites with quantitative data are labelled with (Q) to differentiate them from those with semi-quantitative data. *p < 0.05, **p < 0.01, ***p < 0.001

Statistical analysis with logistic regression found that nine out of 568 serum metabolites were negatively associated and 25 metabolites were positively associated with progression to type 2 diabetes, after controlling for FDR (*q* < 0.05). All of these metabolites were associated with progression to type 2 diabetes independent of fasting glucose levels at baseline, physical activity and the future incidence of CVD (ESM Fig. 2a, b). Sixteen of the metabolite associations were significant even after accounting for risk factors such as age, sex, BMI, family history and fasting insulin (Fig. 1, Table 2) or glucose (ESM Fig. 2a, b) level at baseline.

**Table 2:** Metabolites associated with progression to type 2 diabetes at FDR *q* < 0.05

Metabolite	OR (95% CI)^a^	*p* value^a^
Glucose	1.83 (1.40, 2.44)	1.7 × 10^−5^
Mannose	1.76 (1.35, 2.33)	4.3 × 10^−5^
X-16071 (RI: 3616, M: 146.2)	1.65 (1.25, 2.23)	0.00066
α-HB (Q)^b^	1.60 (1.23, 2.13)	0.00077
X-13537 (RI: 5292, M: 295.3)	1.58 (1.21, 2.09)	0.0011
Isoleucine	1.46 (1.08, 2.02)	0.017
X-13452 (RI: 3606, M: 192.2)	1.44 (1.11, 1.90)	0.0079
Valine	1.40 (1.04, 1.90)	0.026
Glutamate (Q)^b^	1.38 (1.04, 1.85)	0.029
X-12844 (RI: 4168, M: 539.3)	1.35 (1.03, 1.78)	0.032
X-12802 (RI: 2731, M: 318.2)	1.30 (1.03, 1.67)	0.029
Trehalose	1.29 (1.02, 1.65)	0.036
Histidine	0.68 (0.52, 0.89)	0.0054
Bilirubin (E,E)	0.62 (0.44, 0.84)	0.0033
Glutamine	0.58 (0.39, 0.82)	0.0046
α-Tocopherol	0.53 (0.34, 0.77)	0.0023

^a^Conditional ORs (accounting for the risk factors of age, sex, BMI, fasting insulin level and family history of type 2 diabetes), 95% CIs and *p* values were calculated using multivariate logistic regression

^b^Metabolites with quantitative data are labelled with (Q) to differentiate them from those with semi-quantitative data

M, mass to charge ratio of the peak; RI, retention index

Reduced levels of glutamine, histidine, α-tocopherol and the (E,E)-isomer of bilirubin at baseline were associated with an increased risk of type 2 diabetes, independent of the risk factors considered. Increased levels of glutamate, α-HB, valine, isoleucine, trehalose and several unknown metabolites were associated with progression to type 2 diabetes independent of the risk factors (Fig. 1).

### The entire metabolomic profile predicts future progression to type 2 diabetes

**Fig. 2:**
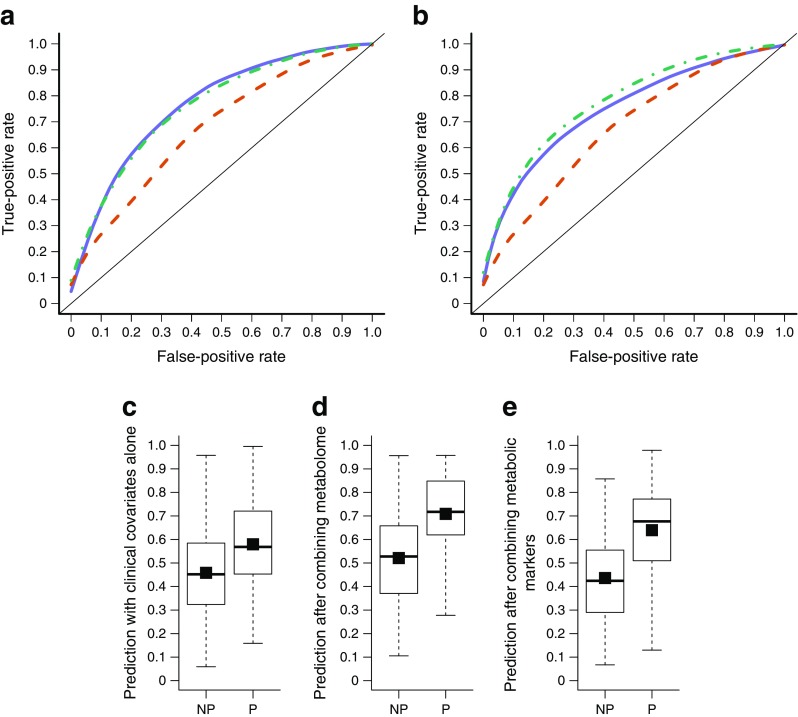
ROC curves of the predictive models based on (**a**) the entire metabolome (i.e. set of 568 metabolites) and (**b**) selected metabolic markers: glucose, mannose, α-HB, X-12063, α-tocopherol, [Hyp3]-BK and X-13435. The mean AUC value obtained with the clinical-only model was 0.68 (95% CI 0.48, 0.86) (red dashed line). (**a**) The metabolome-only model (solid blue line) had a mean AUC of 0.77 (95% CI 0.62, 0.90), while the combined model (green dashed-dot line) had a mean AUC of 0.76 (95% CI 0.59, 0.92). (**b**) The selected metabolic markers (solid blue line) had a mean AUC of 0.75 (95% CI 0.59, 0.89), while the combined model (dashed-dot line) had a mean AUC of 0.78 (95% CI 0.61, 0.92). DS plots of (**c**) the clinical-only model (DS = 0.12), (**d**) the combined model with clinical covariates and 568 metabolites (DS = 0.19) and (**e**) the combined model with clinical covariates and metabolic markers (DS = 0.20). White boxes in the DS plots show the predicted probabilities for progressors (P) and non-progressors (NP), and the black squares inside the boxes show the mean probabilities per group. The IDI was 58% with the entire metabolome and 66.7% with the selected markers

We tested how accurately the metabolome could predict progression to type 2 diabetes by applying a binary classification based on RLS regression. The entire metabolomic profile consisting of 568 serum metabolites predicted progression to type 2 diabetes with an AUC of 0.77 (Fig. 2a). The reference, clinical-only model using RLS regression with only the clinical risk factors of sex, age, BMI, fasting insulin level and family history predicted type 2 diabetes with an AUC of 0.68. The difference in the predictive performance between the metabolomics-only and the clinical-only models was statistically significant (*p* = 0.0009, DeLong’s test). Finally, we combined the clinical risk factors and the metabolomic profile within a joint RLS predictive model. It predicted progression to type 2 diabetes with an AUC of 0.76, a similar accuracy as the metabolomics-only model (*p* = 0.23), but significantly better than the clinical-only model (*p* = 0.005). The clinical-only model resulted in a DS of 0.12. The combined model increased the DS to 0.19, resulting in an IDI of 58% (i.e. 58% improvement in DS) (Fig. 2c, d).

As the fasting glucose level at baseline is a known risk factor for type 2 diabetes, we added this into the clinical model as an additional covariate. Although adding glucose improved the clinical-only model (AUC = 0.70, DS = 0.14), the combined model showed significantly better performance (*p* = 0.023, IDI = 36%; ESM Fig. 3).

An additional clinical reference model that added fasting glucose, total cholesterol, HDL-cholesterol, triacylglycerols, SBP, DBP and waist circumference improved the clinical-only model (AUC = 0.71, DS = 0.15), although the combined model again remained significantly better (*p* = 0.04, IDI = 33%, ESM Fig. 4).

### Specific metabolic biomarkers predict future progression to type 2 diabetes

To better interpret the predictive ability of the metabolome, we sought to identify the key metabolite features required for optimal prediction accuracy by applying GreedyRLS. In order to find the number of features required for optimal prediction performance, we evaluated GreedyRLS by varying the model size from one to 20 features, and thus determined that five metabolites were sufficient for the maximal AUC (ESM Fig. 5).

The five-metabolite signatures selected during 100 repetitions of GreedyRLS predicted type 2 diabetes with an average AUC of 0.75 (Fig. 2b), showing higher predictive accuracy than the reference clinical-only model (AUC = 0.68), although the difference was not significant (*p* = 0.18). However, combining the panel of all selected metabolite features with clinical variables led to the highest predictive performance (AUC = 0.78; see also ESM Results), showing significant improvement over the clinical-only model (*p* = 0.00019; DS = 0.2, IDI = 66.7%; Fig. 2c, e) as well as over the metabolite-only model (*p* = 0.0004). Combining the selected metabolites also significantly improved performance over the additional clinical models, namely the model that contained fasting glucose (*p* = 0.0016, IDI = 43%, ESM Fig. 3) and the model that contained fasting glucose, total cholesterol, HDL-cholesterol, triacylglycerols, SBP, DBP and waist size (*p* = 0.0025, IDI = 40%, ESM Fig. 4) as additional clinical covariates.

**Fig. 3:**
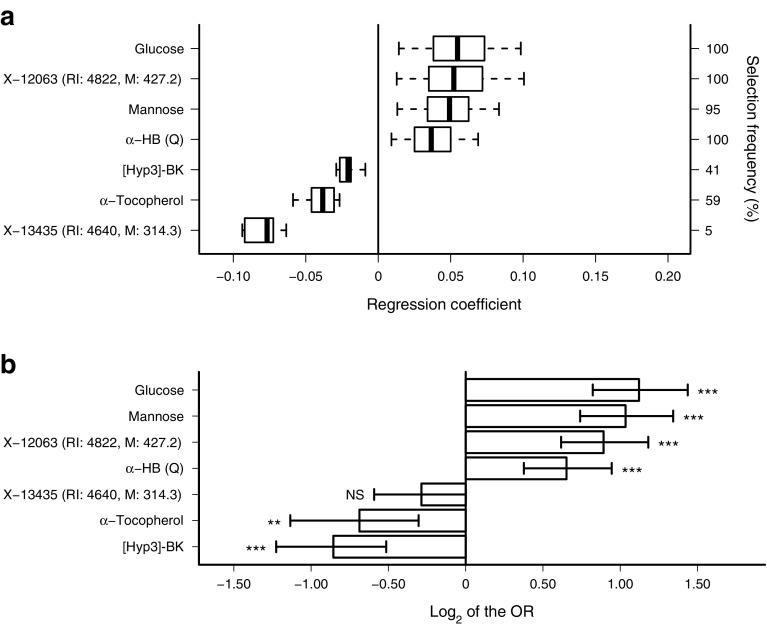
Metabolic markers identified based on 100 repetitions of GreedyRLS. (**a**) Boxes show the spread of regression coefficients of the selected features over the repetitions. The sign of a coefficient indicates whether the marker increased or decreased the risk of type 2 diabetes, and the magnitude indicates the predictive strength of the marker. (**b**) Univariate association of metabolic markers with progression to type 2 diabetes shown as ORs (95% CI). **p < 0.01, ***p < 0.0001

Among the biomarker panel, formed as the union of the metabolic predictors selected during 100 repetitions of GreedyRLS, three metabolites were associated with decreased type 2 diabetes risk: α-tocopherol, bradykinin (BK) hydroxyproline ([Hyp3]-BK) and X-13435; and four were associated with increased risk: α-HB, glucose, mannose and X-12063 (Fig. 3, Table 3). These metabolic predictors, except X-13435, were associated with progression to type 2 diabetes, independent of traditional risk factors as well as of physical activity, use of hypertension medication and future incidence of CVD (ESM Fig. 2c). Mannose showed high correlation with fasting glucose, while X-12063, [Hyp3]-BK and α-HB showed low but statistically significant correlation (ESM Table 2).

**Table 3:** Statistical association of multivariate predictive markers with progression to type 2 diabetes

Metabolite	OR (95% CI)^a^	*p* value^a^
Glucose	2.18 (1.77, 2.71)	7.9 × 10^−13^
Mannose	2.05 (1.67, 2.54)	1.5 × 10^−11^
X-12063 (RI: 4822, M: 427.2)	1.86 (1.53, 2.27)	5.1 × 10^−10^
α-HB (Q)^b^	1.57 (1.3, 1.92)	6.4 × 10^−6^
X-13435 (RI: 4640, M: 314.3)	0.82 (0.66, 1.00)	0.058
α-Tocopherol	0.62 (0.46, 0.81)	0.0011
[Hyp3]-BK	0.55 (0.43, 0.7)	2.3 × 10^−6^

^a^ORs, 95% CIs and *p* values were calculated using logistic regression

^b^Metabolites with quantitative data are labelled with (Q) to differentiate them from those with semi-quantitative data

M, mass to charge ratio of the peak; RI, retention index

**Fig. 4:**
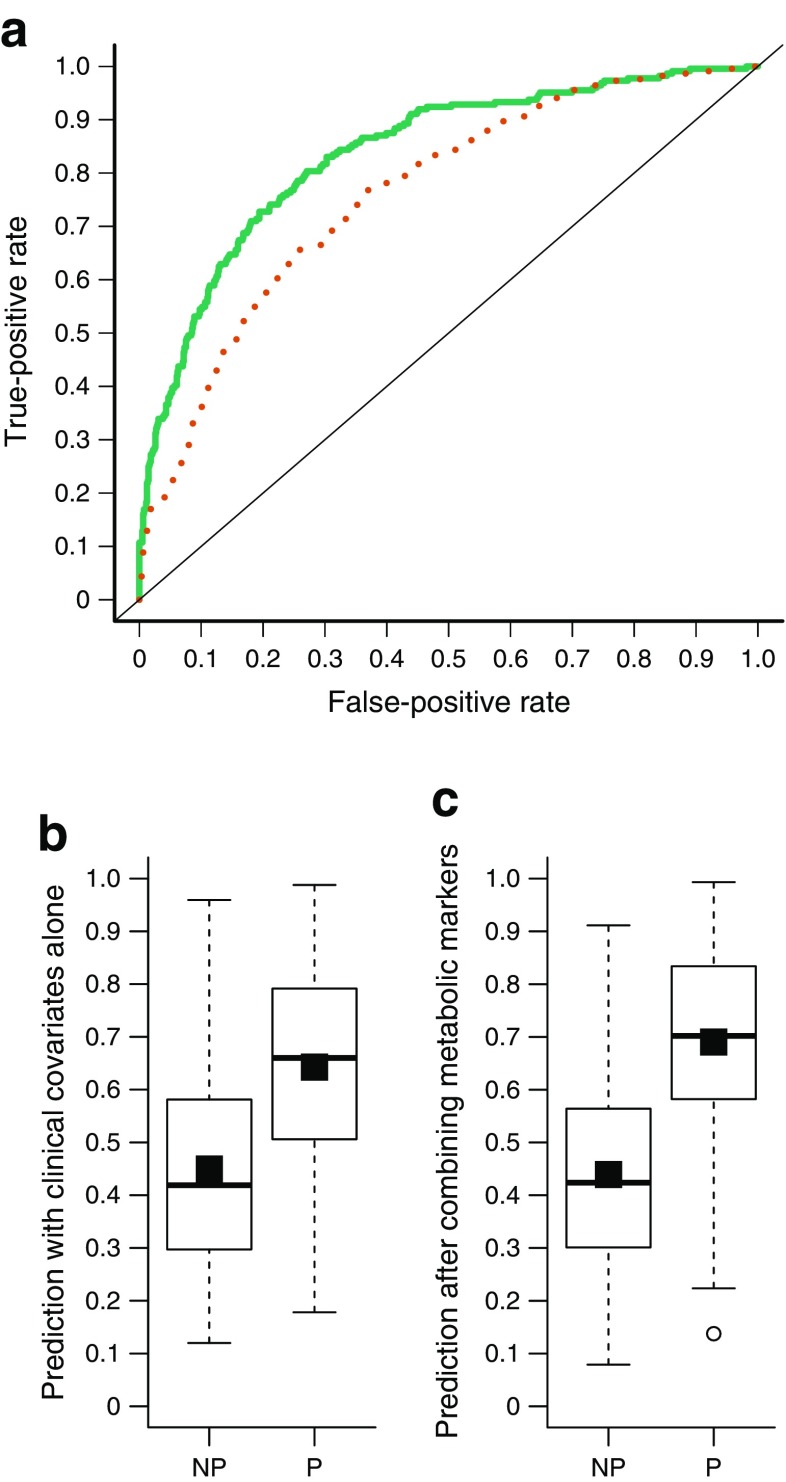
(**a**) ROC curves for the predictive models based on the metabolic markers glucose, mannose, α-HB and α-tocopherol in the DESIR study as an independent validation of the marker panel. The clinical-only model (red dotted line) included the clinical risk factors sex, age, BMI, fasting insulin level and family history of type 2 diabetes, while the combined model (green solid line) included the clinical risk factors and metabolic markers. The mean AUC was 0.76 (95% CI 0.73, 0.80) for the clinical-only model and 0.84 (95% CI 0.81, 0.87) for the combined model. The combined model showed a significant improvement over the clinical-only model (*p* = 5.4 × 10^−7^). DS plots of (**b**) the clinical-only model (DS = 0.19) and (**c**) the combined model (DS = 0.25). White boxes in the DS plots show the predicted probabilities for progressors (P) and non-progressors (NP), and the black squares inside the boxes show the mean probabilities per group. The IDI obtained after adding the metabolic predictors to the clinical-only model was 31.6%

The metabolomics data from the DESIR study included semi-quantitative measurements of four of our seven metabolic markers, namely glucose, mannose, α-HB and α-tocopherol [10]. We used these metabolites and the clinical covariates of sex, age, BMI, family history and fasting insulin measured in the DESIR study samples to predict the progression of these individuals to type 2 diabetes (Fig. 4). The validation AUC values for the clinical-only and combined models were 0.76 and 0.84, respectively, and the selected metabolic markers significantly improved the prediction performance over the clinical-only model (*p* = 5.4 × 10^−7^; IDI = 31.6%).

## Discussion

Using comprehensive metabolomics profiling, we have identified a novel multivariate panel of metabolic markers consisting of glucose, mannose, α-HB, α-tocopherol, [Hyp3]-BK, X-12063 and X-13435, whose concentrations in fasting serum samples predicted future progression to type 2 diabetes in an otherwise healthy, normoglycaemic population, years before the onset of type 2 diabetes (Fig. 3, Table 3). These metabolic markers significantly improved the prediction of progression towards type 2 diabetes, showing the added value of screening metabolites along with clinical risk factors.

Statistical association testing and machine learning-based predictive modelling identified metabolic changes that preceded type 2 diabetes. Statistical tests identified 34 significant metabolites, yet multivariate predictive models required only five metabolites for the optimal prediction of progression to type 2 diabetes. While the metabolite features identified using both approaches are well supported in type 2 diabetes literature, our novel contribution was in systematically assessing the predictive performance of the biomarker panel in type 2 diabetes risk prediction.

### Statistical associations

Histidine, glutamine and the (E,E)-isomer of bilirubin were negatively associated with type 2 diabetes risk independent of clinical risk factors (ESM Fig. 2b). Histidine-mediated suppression of hepatic glucose production has previously been suggested as a potential target for the treatment of type 2 diabetes [25]. In a double-blind placebo-controlled trial in participants with type 2 diabetes, glutamine supplementation yielded positive results [26]. Bilirubin has previously been reported to be negatively correlated with progression to diabetic nephropathy in individuals with type 2 diabetes [27, 28].

Glutamate and trehalose were positively associated with type 2 diabetes risk independent of clinical risk factors (Table 2, Fig. 1, ESM Fig. 2a). Plasma glutamate levels are known to be elevated in several diseases characterised by chronic oxidative stress and inflammation. Furthermore, chronically high extracellular glutamate levels may directly or indirectly contribute to the progressive loss of beta cells in both type 1 and type 2 diabetes [29]. Although trehalose is widely regarded as a safe food ingredient even for individuals with diabetes, trehalose was associated with increased type 2 diabetes risk in this study. Similar results have been previously reported in an African-American population [30].

### Predictive modelling

The machine learning model based on the entire metabolome accurately predicted the future incidence of type 2 diabetes. To derive a more interpretable model, we performed feature selection and identified the most predictive metabolic markers (Fig. 3, Table 3). Combining clinical variables with the selected markers significantly improved the predictive performance over the reference model with clinical variables alone, and led to the model with the highest predictive performance. Even after excluding glucose, the combined predictive model outperformed the clinical reference model that contained fasting glucose (*p* = 0.0066, ESM Fig. 6), showing that the predictive performance of the marker panel was not solely due to glucose. Excluding glucose as well as mannose from the marker panel resulted in a reasonable combined predictive model (AUC = 0.75) that outperformed the reference model (*p* = 0.04), further supporting the value of our novel markers.

Among the biomarkers identified using feature selection (Fig. 3, Table 3), three showed negative associations and four showed positive associations with type 2 diabetes risk. The negatively associated markers were [Hyp3]-BK, α-tocopherol and X-13435 and, to the best of our knowledge, this is the first metabolomics study revealing the value of these markers in predicting type 2 diabetes risk. The positively associated markers were glucose, mannose, α-HB and X-12063. Mechanisms by which mannose is associated with an increased risk of type 2 diabetes independently of glucose have previously been suggested [31]. Identifying the unknown markers X-13435 and X-12063, although non-trivial, may reveal potentially novel pathways associated with progression to type 2 diabetes.

#### BK

BK is a peptide that causes vasodilation associated with BP lowering and protection from CVD [32]. [Hyp3]-BK is a BK analogue in which the third amino acid, proline, is hydroxylated. In this study, [Hyp3]-BK was negatively associated with type 2 diabetes risk independent of CVD risk (*p* = 2.2 × 10^−6^). However, as BK was elevated in progressors, showing an opposite trend (data not shown), we additionally tested the association of the total amount of BK and [Hyp3]-BK with progression. The total BK level was also negatively associated with progression to type 2 diabetes (*p* = 0.004). Diabetes decreases the activity of the kallikrein–kinin system by reducing the synthesis of plasma prekallikrein and hence BK, resulting in endothelial dysfunction [33, 34]. The current study revealed reduced levels of total BK far before the onset of type 2 diabetes, indicating a potential early role for the kallikrein–kinin system or oxidative stress and DNA damage associated with reduced BK in progression to type 2 diabetes. Interestingly, physical exercise improves glucose uptake by skeletal muscle resulting in improved insulin sensitivity, an effect that is partially mediated by an increased BK concentration, suggesting a mechanism by which physical exercise would contribute to the prediction of type 2 diabetes [33, 35–37]. [Hyp3]-BK was associated with type 2 diabetes risk independently of physical activity, antihypertensive medication and CVD (ESM Fig. 2c).

#### α-Tocopherol

A reduction in α-tocopherol, the most biologically active form of vitamin E in humans, was seen in progressors, and it was selected in the biomarker panel. Although observational studies have previously indicated a protective effect of vitamin E supplementation on glycaemic control in type 2 diabetes, randomised controlled trials have not confirmed the effect [38–40].

#### The unknowns (X-13435 and X-12063)

The unknown metabolite X-12063 showed a strong association with progression towards type 2 diabetes, and was selected in the biomarker panel (Fig. 3). Although its identity is currently unknown, this metabolite has previously been noted to be significantly associated with insulin resistance and glucose intolerance [14]. X-13435, which was not identified in earlier metabolomics studies, did not show univariate statistical association, but predicted type 2 diabetes risk jointly with the other markers.

All of the metabolic markers were associated with progression to type 2 diabetes independently of fasting glucose (ESM Fig. 2c). Except for X-13435, they were all associated with progression to type 2 diabetes independently of CVD, physical activity and use of hypertension medication. While mannose was highly correlated with fasting glucose, [Hyp3]-BK and X-12063 showed statistically significant yet low correlations. Similarly, α-HB showed a relatively low correlation, although statistically significant, with fasting glucose (ESM Table 2).

Taken together, the statistical analysis and predictive modelling identified a variety of known metabolic changes associated with progression to type 2 diabetes (ESM Fig. 2). In agreement with a recent meta-analysis [9], our study identified changes in BCAAs (valine and isoleucine), amino acids (histidine, glutamine and glycine), sugars (glucose and mannose) and other metabolites (glutamate, α-HB and L-GPC).

### Benchmarking of the predictive markers

According to our benchmarking results, our new biomarker panel performed better than previously published metabolic markers, namely α-HB and L-GPC [14, 15] and amino acids [13]. α-HB was associated with increased insulin resistance and glucose intolerance, whereas L-GPC was protective [14, 15]. Predictive modelling with α-HB and L-GPC revealed a high predictive performance (AUC = 0.72; ESM Fig. 7), when combined with clinical covariates.

BCAAs are associated with insulin resistance, and the combination of isoleucine and the amino acids tyrosine and phenylalanine has been reported to predict the risk of type 2 diabetes 12 years before disease onset [13]. Consistent with previous studies, joint modelling of isoleucine, tyrosine, phenylalanine and clinical covariates showed high predictive performance (AUC = 0.71; ESM Fig. 7). As these amino acids are consistently found in many studies of prediabetes (i.e. impaired glucose tolerance, impaired fasting glucose, insulin resistance or impaired insulin sensitivity) and type 2 diabetes [9], they may have high potential for routine use as predictive biomarkers, and further translational research is needed to facilitate their clinical use.

### Limitations of the study

Distinct predictive signatures may be discovered in different studies due to differences in the metabolites measured, or differences in the genetic and environmental background of the study population. The relatively large overlap of our biomarker panel with known markers of type 2 diabetes, however, suggests that our results are robust and stable. Replication of our findings in the DESIR data also shows that the predictive model trained using Finnish individuals generalised to independent French individuals, despite the potential variability due to the demographic difference.

α-HB was quantitatively measured in the BPS dataset, but using an untargeted platform in the DESIR dataset. Moreover, three of the metabolic markers, [Hyp3]-BK, X-12063 and X-13435, were not measured in the DESIR study. Despite these differences, we confirmed the high predictive performance and added predictive value of the selected metabolic markers in the independent study.

## Conclusions

Progressors and non-progressors have different metabolic profiles years before they develop overt type 2 diabetes. In this study, a combination of known markers such as glucose, mannose and α-HB, and novel markers such as α-tocopherol, [Hyp3]-BK, X-12063 and X-13435, was found to accurately predict progression to type 2 diabetes. Interestingly, the negative association of [Hyp3]-BK with progression to type 2 diabetes highlights a possible mechanism by which interventions such as exercise could contribute to the prevention of type 2 diabetes.

## Electronic supplementary material

ESM (PDF 4635 kb)

## Abbreviations

BCAA: Branched chain amino acid
BK: Bradykinin
BPS: Botnia Prospective Study
CVD: Cardiovascular disease
DESIR: Data from an Epidemiological Study on the Insulin Resistance Syndrome
DBP: Diastolic BP
DS: Discrimination slope
FDR: False discovery rate
GreedyRLS: Greedy feature selection for regularised least squares
α-HB: α-Hydroxybutyrate
[Hyp3]-BK: Bradykinin hydroxyproline
IDI: Integrated discrimination improvement
L-GPC: 1-Linoleoyl glycerophosphocholine
RISC: Relationship of Insulin Sensitivity to Cardiovascular Risk
RLS: Regularised least squares
ROC: Receiver operating characteristic
SBP: Systolic BP
UHPLC: Ultra-HPLC

## References

[1] NCD Risk Factor Collaboration (NCD-RisC) (2016). Worldwide trends in diabetes since 1980: a pooled analysis of 751 population-based studies with 4.4 million participants. Lancet.

[2] Wishart DS (2016). Emerging applications of metabolomics in drug discovery and precision medicine. Nat Rev Drug Discov.

[3] Goodacre R, Vaidyanathan S, Dunn WB, Harrigan GG, Kell DB (2004). Metabolomics by numbers: acquiring and understanding global metabolite data. Trends Biotechnol.

[4] Bain JR, Stevens RD, Wenner BR, Ilkayeva O, Muoio D, Newgard CB (2009). Metabolomics applied to diabetes research: moving from information to knowledge. Diabetes.

[5] Wang-Sattler R, Yu Z, Herder C (2012). Novel biomarkers for pre-diabetes identified by metabolomics. Mol Syst Biol.

[6] Suhre K, Meisinger C, Döring A (2010). Metabolic footprint of diabetes: a multiplatform metabolomics study in an epidemiological setting. PLoS One.

[7] Floegel A, Stefan N, Yu Z (2013). Identification of serum metabolites associated with risk of type 2 diabetes using a targeted metabolomic approach. Diabetes.

[8] Klein MS, Shearer J (2016). Metabolomics and type 2 diabetes: translating basic research into clinical application. J Diabetes Res.

[9] Guasch-Ferré M, Hruby A, Toledo E (2016). Metabolomics in prediabetes and diabetes: a systematic review and meta-analysis. Diabetes Care.

[10] Yengo L, Arredouani A, Marre M (2016). Impact of statistical models on the prediction of type 2 diabetes using non-targeted metabolomics profiling. Mol Metab.

[11] Menni C, Fauman E, Erte I (2013). Biomarkers for type 2 diabetes and impaired fasting glucose using a nontargeted metabolomics approach. Diabetes.

[12] Drogan D, Dunn WB, Lin W (2015). Untargeted metabolic profiling identifies altered serum metabolites of type 2 diabetes mellitus in a prospective, nested case control study. Clin Chem.

[13] Wang TJ, Larson MG, Vasan RS (2011). Metabolite profiles and the risk of developing diabetes. Nat Med.

[14] Gall WE, Beebe K, Lawton KA (2010). α-Hydroxybutyrate is an early biomarker of insulin resistance and glucose intolerance in a nondiabetic population. PLoS One.

[15] Ferrannini E, Natali A, Camastra S (2013). Early metabolic markers of the development of dysglycaemia and type 2 diabetes and their physiological significance. Diabetes.

[16] Lyssenko V, Almgren P, Anevski D (2005). Predictors of and longitudinal changes in insulin sensitivity and secretion preceding onset of type 2 diabetes. Diabetes.

[17] Evans AM, DeHaven CD, Barrett T, Mitchell M, Milgram E (2009). Integrated, nontargeted ultrahigh performance liquid chromatography/electrospray ionization tandem mass spectrometry platform for the identification and relative quantification of the small-molecule complement of biological systems. Anal Chem.

[18] Benjamini Y, Hochberg Y (1995). Controlling the false discovery rate: a practical and powerful approach to multiple testing. J R Stat Soc Ser B Methodol.

[19] Pahikkala T, Okser S, Airola A, Salakoski T, Aittokallio T (2012). Wrapper-based selection of genetic features in genome-wide association studies through fast matrix operations. Algorithm Mol Biol.

[20] Krstajic D, Buturovic LJ, Leahy DE, Thomas S (2014). Cross-validation pitfalls when selecting and assessing regression and classification models. J Cheminform.

[21] DeLong ER, DeLong DM, Clarke-Pearson DL (1988). Comparing the areas under two or more correlated receiver operating characteristic curves: a nonparametric approach. Biometrics.

[22] Sing T, Sander O, Beerenwinkel N, Lengauer T (2005). ROCR: visualizing classifier performance in R. Bioinformatics.

[23] Robin X, Turck N, Hainard A (2011). pROC: an open-source package for R and S+ to analyze and compare ROC curves. BMC Bioinforma.

[24] Pencina MJ, D’Agostino RB, D’Agostino RB, Vasan RS (2008). Evaluating the added predictive ability of a new marker: from area under the ROC curve to reclassification and beyond. Stat Med.

[25] Kimura K, Nakamura Y, Inaba Y (2013). Histidine augments the suppression of hepatic glucose production by central insulin action. Diabetes.

[26] Mansour A, Mohajeri-Tehrani MR, Qorbani M, Heshmat R, Larijani B, Hosseini S (2015). Effect of glutamine supplementation on cardiovascular risk factors in patients with type 2 diabetes. Nutrition.

[27] Hull TD, Agarwal A (2014). Bilirubin: a potential biomarker and therapeutic target for diabetic nephropathy. Diabetes.

[28] Riphagen IJ, Deetman PE, Bakker SJL (2014). Bilirubin and progression of nephropathy in type 2 diabetes: a post hoc analysis of RENAAL with independent replication in IDNT. Diabetes.

[29] Davalli AM, Perego C, Folli FB (2012). The potential role of glutamate in the current diabetes epidemic. Acta Diabetol.

[30] Yu B, Zheng Y, Alexander D, Morrison AC, Coresh J, Boerwinkle E (2014). Genetic determinants influencing human serum metabolome among African Americans. PLoS Genet.

[31] Lee S, Zhang C, Kilicarslan M (2016). Integrated network analysis reveals an association between plasma mannose levels and insulin resistance. Cell Metab.

[32] Palkhiwala SA, Frishman WH, Warshafsky S (2001). Bradykinin for the treatment of cardiovascular disease. Heart Dis.

[33] Simões HG, Asano RY, Sales MM (2013). Type 2 diabetes elicits lower nitric oxide, bradykinin concentration and kallikrein activity together with higher DesArg(9)-BK and reduced post-exercise hypotension compared to non-diabetic condition. PLoS One.

[34] Stabler T, Kenjale A, Ham K, Jelesoff N, Allen J (2010). Potential mechanisms for reduced delivery of nitric oxide to peripheral tissues in diabetes mellitus. Ann N Y Acad Sci.

[35] Kolodka T, Charles ML, Raghavan A (2014). Preclinical characterization of recombinant human tissue kallikrein-1 as a novel treatment for type 2 diabetes mellitus. PLoS One.

[36] Asano RY, Sales MM, Browne RA (2014). Acute effects of physical exercise in type 2 diabetes: a review. World J Diabetes.

[37] Taguchi T, Kishikawa H, Motoshima H (2000). Involvement of bradykinin in acute exercise-induced increase of glucose uptake and GLUT-4 translocation in skeletal muscle: studies in normal and diabetic humans and rats. Metab Clin Exp.

[38] Kataja-Tuomola MK, Kontto JP, Männistö S, Albanes D, Virtamo JR (2010). Effect of alpha-tocopherol and beta-carotene supplementation on macrovascular complications and total mortality from diabetes: results of the ATBC study. Ann Med.

[39] Kataja-Tuomola M, Sundell JR, Männistö S (2008). Effect of α-tocopherol and β-carotene supplementation on the incidence of type 2 diabetes. Diabetologia.

[40] Xu R, Zhang S, Tao A, Chen G, Zhang M (2014). Influence of vitamin E supplementation on glycaemic control: a meta-analysis of randomised controlled trials. PLoS One.

