# Supplementary material for: Early metabolic markers identify potential targets for the prevention of type 2 diabetes
Source: Diabetologia. 2017 Jun 8;60(9):1740–50. doi: 10.1007/s00125-017-4325-0 (PMC5552834; doi:10.1007/s00125-017-4325-0)
Supplement: Supplementary file 1 — (PDF 4635 kb) [file 125_2017_4325_MOESM1_ESM.pdf]

Electronic Supplementary Material (ESM)

Early metabolic markers identify potential targets for the prevention of type 2 diabetes

Gopal Peddinti, Jeff Cobb, Loic Yengo, Philippe Froguel, Jasmina Kravić, Beverley Balkau, Tiinamaija Tuomi, Tero Aittokallio and Leif Groop

Abbreviations and acronyms used in ESM

Alpha-hydroxybutyrate	α-HB
Area under the receiver operating characterstic curve	AU-ROC
Fasting glucose levels at the baseline	bGlu
Fasting insulin levels at the baseline	bIns
95% confidence interval	CI
Cardiovascular diseases at any visit during follow-up period	CVD
Discrimination slope	DS
Family history of T2D	FH
Greedy feature selection algorithm for RLS	GreedyRLS
Hypertension medication (Diuretics, Beta blockers, Calcium blockers, ACE inhibitors, AT2 receptor inhibitors, or other blood pressure medication)	HT Med
Bradykinin hydroxyproline	[Hyp3]-BK
Integrated discrimination improvement	IDI
Logarithm of the odds ratio	log odds
Physical activity	PhyAct
Regularized least squares approach	RLS
Type 2 diabetes	T2D

ESM Methods

Metabolomics

The biochemical profiling protocols and experimental procedures applied for sample preparation, untargeted and targeted metabolomics, and data preprocessing applied in the current study are similar to the protocols explained in detail previously [1, 2], and were performed by Metabolon Inc. (Durham, NC, USA). Here we present the methods in brief, for completeness.

Prior to extraction, samples were stored at -80 °C. On the day of extraction, samples were thawed on ice and 100 µl was extracted using an automated MicroLab STARH system (Hamilton Company, Salt Lake City, UT, USA). The samples were extracted using a single extraction with 400 ml of methanol, containing the recovery standards: tridecanoic acid, fluorophenylglycine, chlorophenylalanine and d6-cholesterol. The solvent extraction step was performed by shaking for two minutes using a Geno/Grinder 2000 (Glen Mills Inc., Clifton, NJ, USA). After extraction, the sample was centrifuged and supernatant removed using the MicroLab STARH robotics system. The extract supernatant was split into four equal aliquots: two for UHPLC/MS, one for GC/MS and one reserve aliquot. Aliquots were placed on a TurboVapH (Zymark/Biotage LLC, Charlotte, NC, USA) to remove solvent, and dried under vacuum overnight. Samples were maintained at 4 °C throughout the extraction process. For UHPLC/MS analysis, extract aliquots were reconstituted in either 0.1% formic acid for positive ion UHPLC/MS, or 6.5 mmol/l ammonium bicarbonate pH 8.0 for negative ion UHPLC/MS. For GC/MS analysis, aliquots were derivatized using equal parts N,O-bistrimethylsilyl-trifluoroacetamide and a solvent mixture of acetonitrile:dichlor-omethane:cyclohexane (5:4:1) with 5% triethylamine at 60 °C for 1 hour. The derivatization mixture also contained a series of alkylbenzenes for use as retention time markers.

Global metabolomic profiling was performed using multiple platforms, ultra high performance liquid chromotography (UHPLC) and gas chromatography (GC), coupled with mass spectrometry (MS) technology. A broad array of molecules covering many metabolite classes, including amino

acids, lipids, carbohydrates, was measured in serum samples collected after subjects fasted overnight (10–12 hours). The non-targeted metabolite profiling was done using single sample extraction followed by protein precipitation to recover a diverse range of molecules including polar and hydrophobic molecules.

UHPLC/MS was carried out using a Waters Acquity UHPLC (Waters Corporation, Milford, MA, USA) coupled to an LTQ mass spectrometer (Thermo Fisher Scientific Inc., Waltham, MA, USA) equipped with an electrospray ionization source (ESI). Two separate UHPLC/MS injections were performed on each sample: one optimized for positive ions (ESI+) and the other for negative ions (ESI-). The ESI+ analyses were performed first, followed by ESI- analyses.

For absolute quantitation, metabolites were analyzed by isotope-dilution ultra-high-performance liquid chromatographic tandem mass spectroscopy (UHPLC-MS/MS) assay. 50 ml of EDTA plasma samples were spiked with internal standard solution and subsequently subjected to protein precipitation by mixing with 250 ml of methanol. Following centrifugation, aliquots of clear supernatant were injected onto an UHPLC-MS-MS system, consisting of a Thermo TSQ Quantum Ultra Mass Spectrometer and a Waters Acquity UHPLC system equipped with a column manager module and three different columns. Each sample was analyzed using three different chromatographic systems to cover the various analytes. Quantitation was performed based on the area ratios of analyte and internal standard peaks using a weighted linear least squares regression analysis generated from fortified calibration standards in an artificial matrix, prepared immediately prior to each run.

Metabolites were identified by automated comparison of spectra to a chemical standard library of experimentally derived spectra. Identification of known molecules was based on comparison with library entries of purified authentic chemical standards. Missing values were imputed using the minimum non-missing measurement for each metabolite (peak).

ESM Results

Additional blinded validation of metabolic marker panel within Botnia cohort

In addition to estimating the predictive performance of the models using a rigorous nested cross-validation approach, we identified 226 out of 543 subjects with missing measurements in one or more clinical covariates, and utilized them as a blind test cohort, in order to provide a more detailed assessment of the selected biomarker panel within the BPS cohort. Five-metabolite panels trained with 317 subjects showed an AU-ROC of 0.67 in the repeated nested CV and an AU-ROC of 0.79 in the blinded test set, providing an additional independent validation of the selected markers.

Wang et al. [3] reported the predictive performance of their amino acid marker panel in low (c-statistic = 0.65) and high-risk populations (c-statistic = 0.8), containing 50% and 32% progressors, respectively. Interestingly, our independent training and test groups represented similar high and low-risk groups, with 47% and 8% progressors, respectively. Hence, with AU-ROC of 0.67 and 0.79 in high and low-risk groups, respectively, our markers show similar performance even in the low-risk group that contains much lower percentage of progressors, almost identical to global T2D prevalence of 8% in adults.

ESM Tables

ESM Table 1

Clinical characteristics of subjects from D.E.S.I.R. included in this study. The D.E.S.I.R. study samples were used for an independent validation of the predictive models trained based on the Botnia study.

Factor		Total population	Non-progressor	Progressor	P-value
N		1,044	813	231	NA
Sex	Male	546	394	151	6.9×10^{-6}
	Female	499	419	80	
Age (years)[a]		48.18 ± 0.31	47.29 ± 0.35	51.31 ± 0.61	7.2×10^{-8}
BMI (kg/m^2)[a]		25.08 ± 0.12	24.31 ± 0.12	27.79 ± 0.29	7.8×10^{-34}
Fasting glucose (mmol/l)[a]		5.38 ± 0.01	5.23 ± 0.01	5.92 ± 0.04	7.6×10^{-70}
Fasting insulin (pmol/l)[a]		49.3 ± 0.98	43.4 ± 0.81	70.1 ± 3.04	1.9×10^{-31}
T2D family history (FH)	No	827	657	170	0.02
	Yes	217	156	61	

[a]Data presented as mean ± standard error of mean. Differences tested using Student's t-test.

ESM Table 2

Correlation of the selected metabolic markers with fasting glucose based on the Botnia study.

Metabolite	Pearson correlation coefficient	*p* value
Mannose	0.66	4.4E-69
X - 12063	0.18	1.5E-05
Alpha-Hydroxybutyrate (Q)	0.09	0.04
X - 13435	0.02	0.64
Alpha-Tocopherol	-0.01	0.77
Bradykinin hydroxyproline	-0.23	3.1E-08

ESM figures

ESM Fig. 1

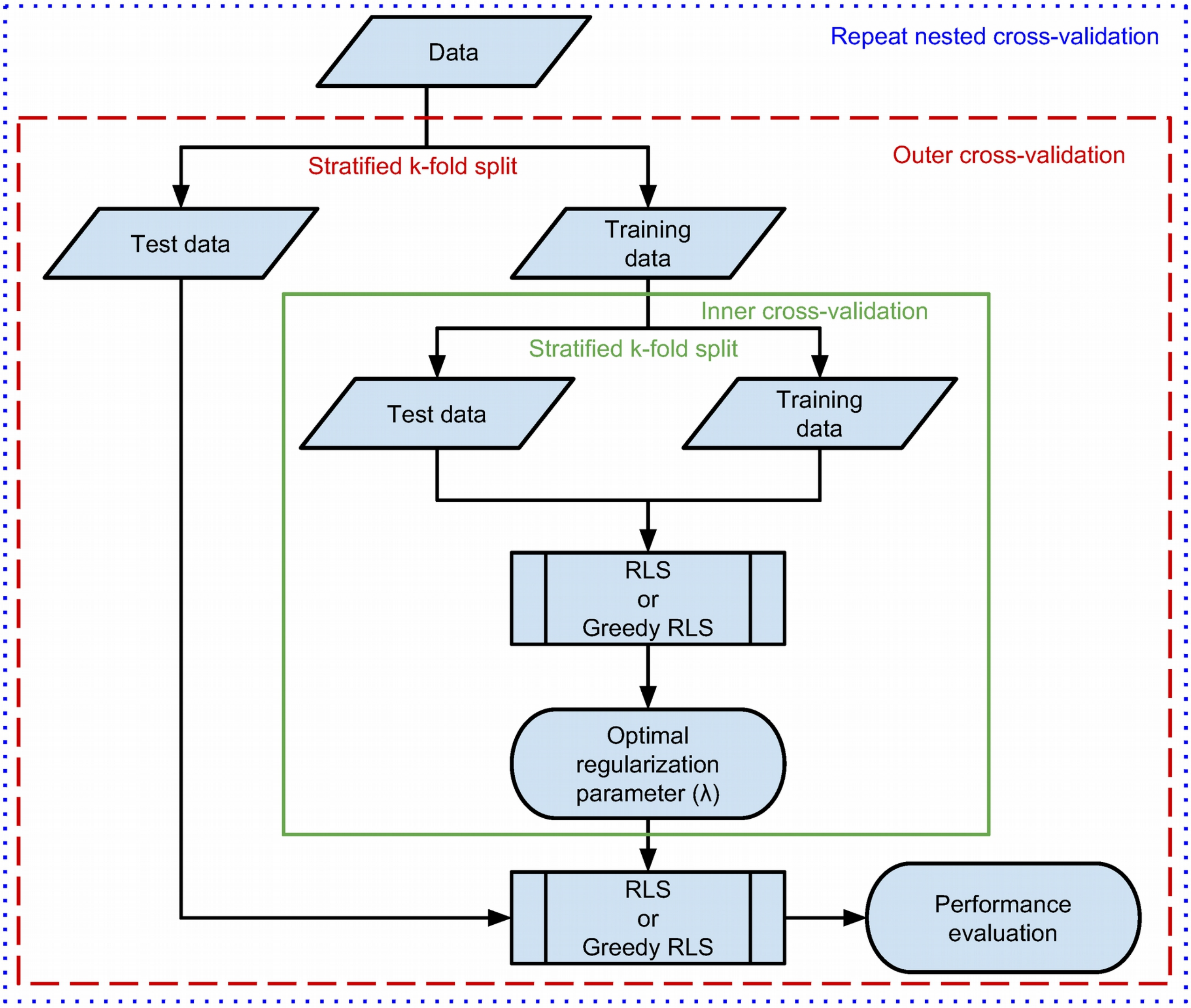

Flow chart of the repeated nested cross-validation procedure applied to learning the predictive models and to evaluating their predictive performance. The inner cross-validation, enclosed by green continuous rectangle, selects the optimal regularization parameter. The outer cross-validation, enclosed by red dashed rectangle, learns the coefficients in the regression model and evaluates the model performance. We performed 10-fold outer and 10-fold inner cross-validation in the model building, and repeated the procedure 100 times (the box enclosed by dotted blue rectangle) to get an average estimate of the performance.

ESM Fig. 2

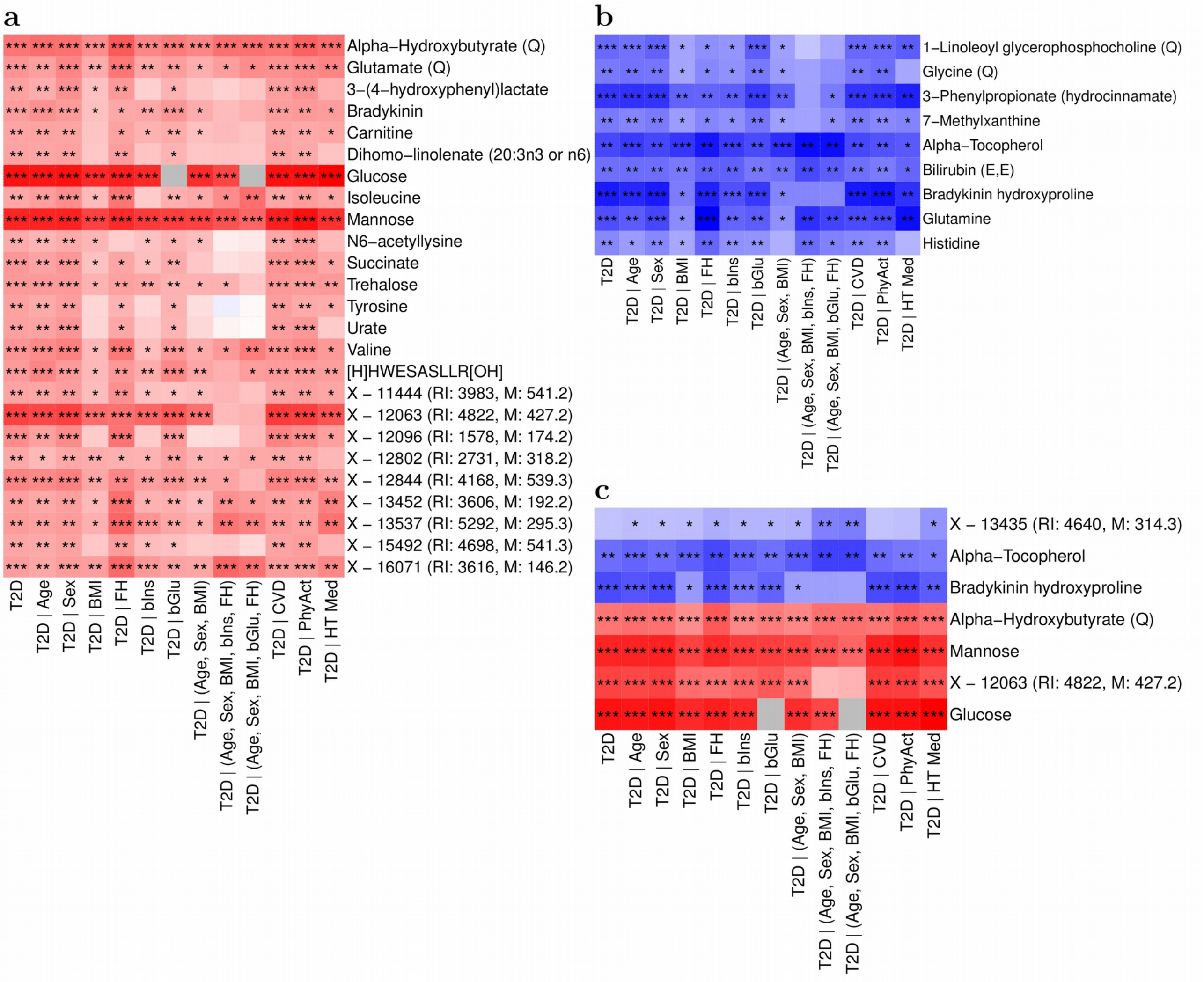

Metabolites positively and negatively associated with progression to T2D according to univariate testing in sub-figures **(a)** and **(b)** respectively, and metabolic markers selected using machine learning in **(c)**. Logarithm of the odds ratio (log odds, first column of the heatmaps) for progression versus non-progression is shown in a color gradient from blue (negative) to white (zero) to red (positive). Figure shows only those metabolites associated with T2D progression at FDR Q < 0.05. The remaining columns in the heatmaps show the conditional log odds after adjusting for the risk factors (Age, Sex, BMI, FH, bIns, bGlu, CVD, PhyAct, HT Med). Asterisks indicate the significance of the log odds or the conditional log odds: *, $p < 0.05$; **, $p < 0.01$; ***, $p < 0.0001$. Metabolites detected with targeted quantitative platform are marked with the suffix '(Q)'. Glucose was excluded from the statistical tests when adjusting for the fasting glucose, hence showing the missing statistic (grey) in (a) and (c) panels.

ESM Fig. 3

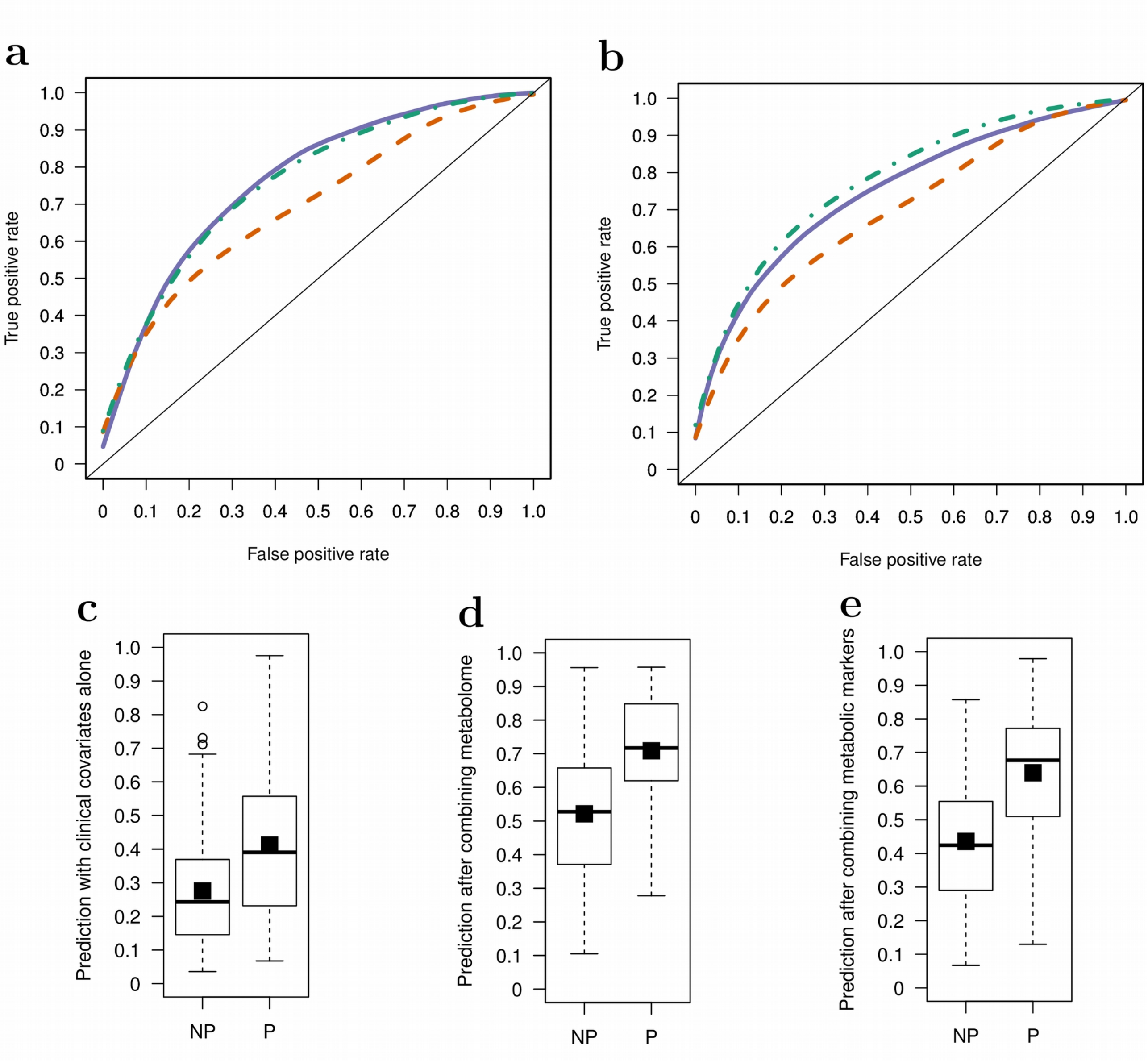

Effect of adding fasting glucose into the clinical-only predictive model (dashed line; mean and CI of AU-ROC, 0.70 and (0.50, 0.88)) in comparison with the predictive models based on **(a)** the entire metabolome including the glucose metabolite (solid line, metabolites-only model, AU-ROC 0.77 (0.62, 0.90); dashed-dot line, combined model, AU-ROC 0.76 (0.59, 0.92); combined *vs* clinical-only, p = 0.023). and **(b)** metabolic marker panel (i.e. glucose, mannose, α-HB, X-12063, α-tocopherol, [Hyp3]-BK, and X-13435) (solid line, metabolites-only model, AU-ROC 0.75 (0.59, 0.89); dashed-dot line, combined model, AU-ROC 0.78 (0.61, 0.92); combined *vs* clinical-only, p = 0.0016). The clinical reference model was built using age, sex, BMI, FH, bIns, and bGlu. The combined *metabolites* + *clinical* models included the clinical covariates: age, sex, BMI, FH, and bIns (i.e. bGlu was not included). The discrimination slope plots of **(c)** *clinical-only* (DS = 0.14), **(d)** *clinical* + *metabolome* (DS = 0.19; IDI = 0.05 showing 35.7% improvement in DS over clinical-only) and **(e)** *clinical* + *selected metabolic markers* (DS = 0.20; IDI = 0.06 showing 42.9% improvement in DS over clinical-only) models. Thus, the *metabolites* + *clinical* models performed significantly better than clinical-only model.

ESM Fig. 4

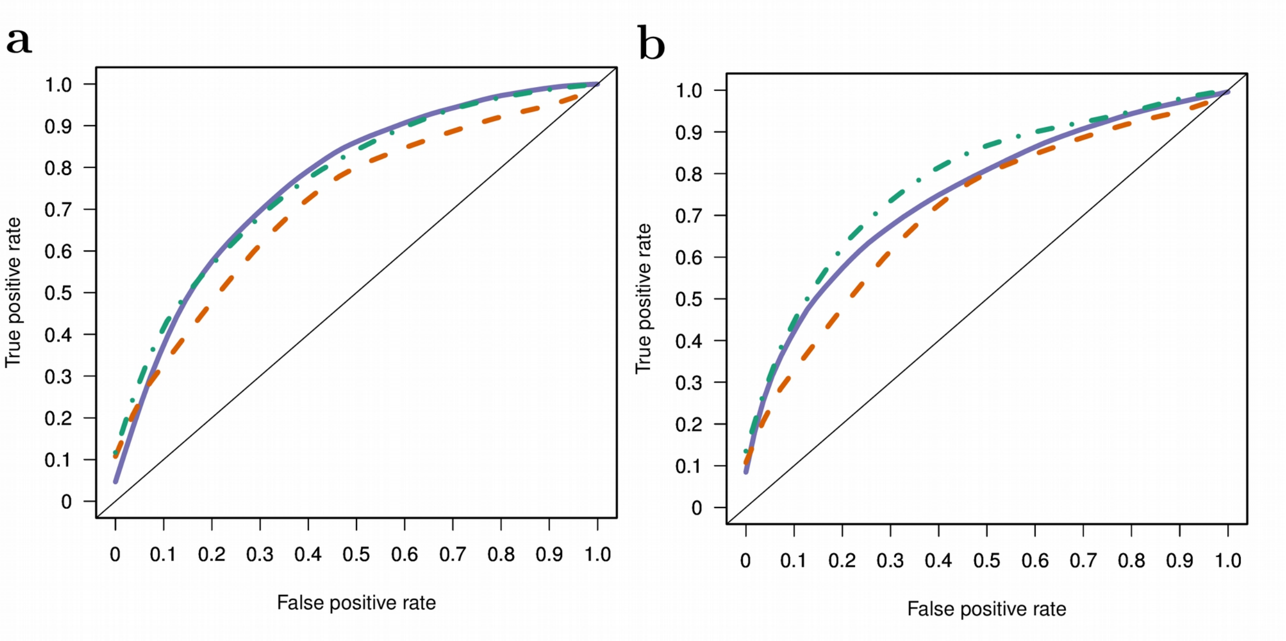

The clinical reference model containing age, sex, BMI, fasting insulin, fasting glucose, family history of type 2 diabetes, waist size, systolic and diastolic blood pressures, total cholesterol, HDL cholesterol, and triacylglycerols (dashed line, AU-ROC 0.71 (0.49, 0.90)) and the assessment of added value of the metabolic markers. ROC curves comparing the effect of **(a)** adding the entire metabolome (excluding glucose metabolite) (solid line, metabolites-only model, 0.77 (0.62, 0.90); dashed-dot line, combined model, 0.77 (0.57, 0.93); combined vs clinical-only, p = 0.04) and **(b)** metabolic marker panel excluding glucose metabolite (i.e. mannose, α-HB, X-12063, α-tocopherol, [Hyp3]-BK, and X-13435) (solid line, metabolites-only model, 0.75 (0.59, 0.89); dashed-dot line, combined model, 0.79 (0.59, 0.94); combined vs clinical-only, p = 0.0025).

ESM Fig. 5

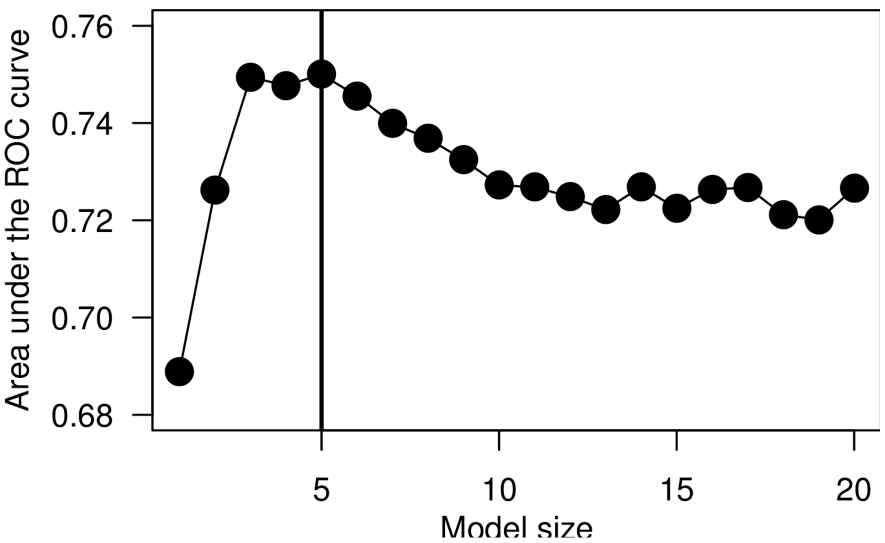

The optimal number of metabolic biomarkers was selected by varying the model size parameter in GreedyRLS between 1 and 20 features, repeating the nested cross-validation 20 times, and calculating the average AU-ROC for each model size. The maximum AU-ROC was obtained with five metabolites.

ESM Fig. 6

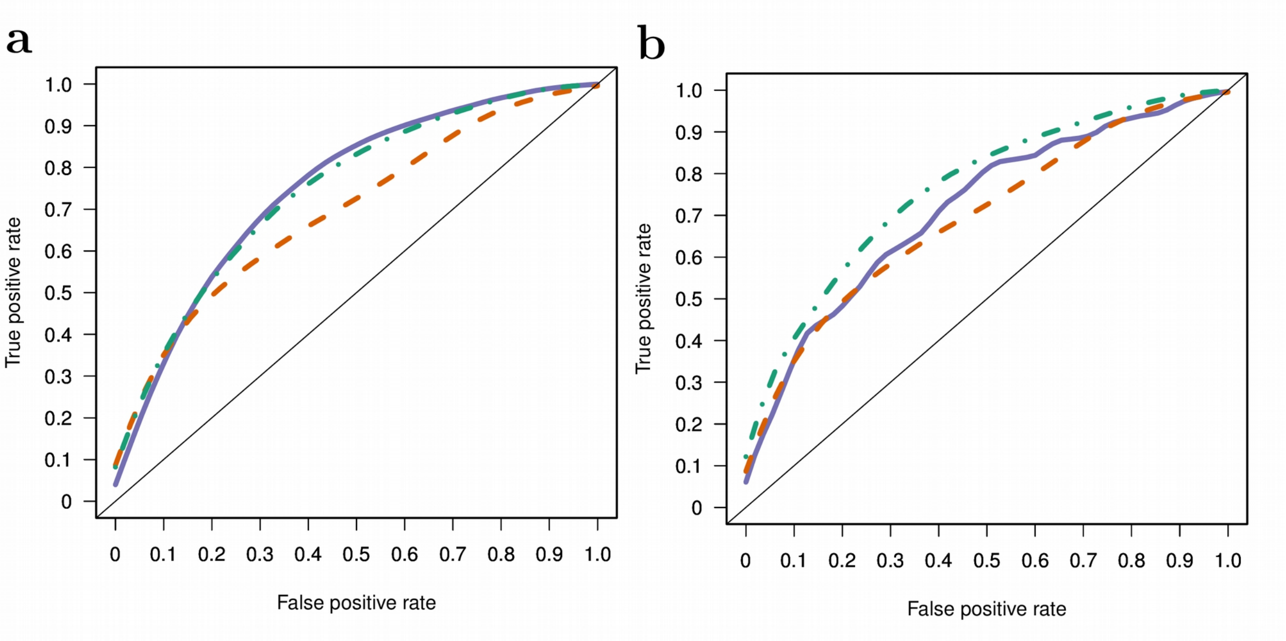

Effect of removing glucose from the metabolite-based predictive models in comparison with clinical reference model that includes fasting glucose (dashed line, AU-ROC 0.70 (0.50, 0.88)). **(a)** Models with the entire metabolome except the glucose metabolite (solid line, metabolites-only, AU-ROC 0.75 (0.62, 0.87); dashed-dot line, combined model, AU-ROC 0.75 (0.57, 0.91); combined vs clinical-only, p = 0.048) and **(b)** the models with the metabolic marker panel excluding the glucose metabolite (i.e. mannose, α-HB, X-12063, α-tocopherol, [Hyp3]-BK, and X-13435) (solid line, metabolites-only, AU-ROC 0.72 (0.53, 0.88); dashed-dot line, combined model, AU-ROC 0.77 (0.57, 0.92); combined vs clinical-only, p = 0.0066). The clinical reference model was built using age, sex, BMI, FH, bIns, and bGlu. The combined *metabolites* + *clinical* models included the clinical covariates: age, sex, BMI, FH, and bIns (i.e. bGlu was not included). The *metabolites* + *clinical* models performed significantly better than clinical only model (p = 0.048, with the entire metabolome; p = 0.0066 with marker panel).

ESM Fig. 7

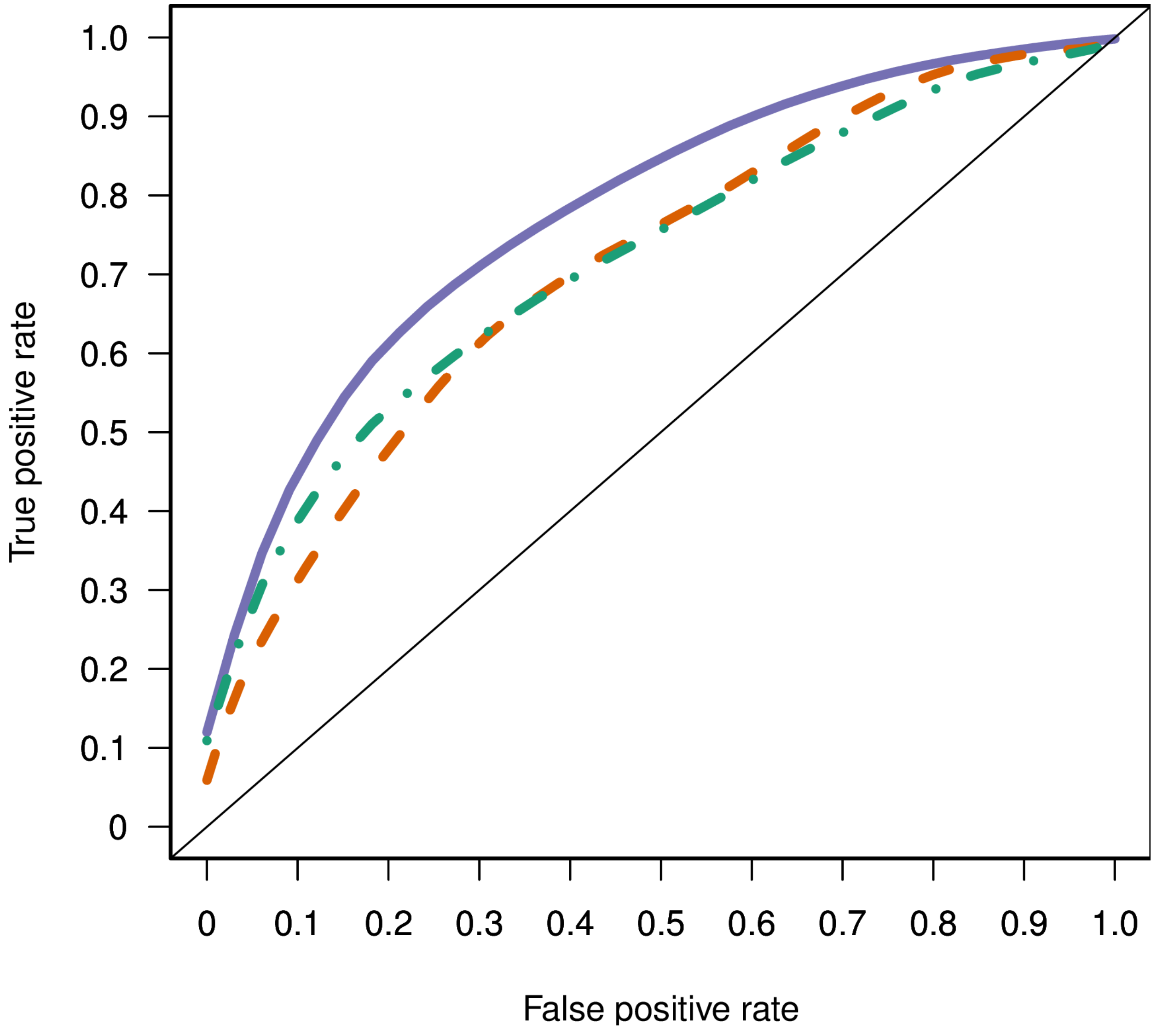

Comparison of the metabolic markers found in the current study (*new markers;* solid line, AU-ROC 0.78 (0.61, 0.92)) with the previously established markers from Wang et al., [3] (dashed line, AU-ROC 0.71 (0.54, 0.86)) and Ferrannini et al., [4] (dashed-dot line, 0.72 (0.51, 0.89)),referred to as *Wang markers* and *Ferrannini markers,* respectively. The *new markers* model indcluded age, sex, BMI, bIns, and FH as clinical covariates. In accordance with the original studies, the *Wang markers* model included age, sex, BMI, and bGlu, and the *Ferrannini markers* model included age, sex, BMI, bGlu, and FH. The ROC curves were constructed using repeated nested cross-validation. Predictive performance of the *new markers* was higher than *Wang markers* (p = 0.0038) and *Ferrannini markers* (p = 0.005).

ESM References

1. Gall WE, Beebe K, Lawton KA, et al. (2010) alpha-hydroxybutyrate is an early biomarker of insulin resistance and glucose intolerance in a nondiabetic population. PLoS ONE 5:e10883.

2. Evans AM, DeHaven CD, Barrett T, et al. (2009) Integrated, nontargeted ultrahigh performance liquid chromatography/electrospray ionization tandem mass spectrometry platform for the identification and relative quantification of the small-molecule complement of biological systems. Anal Chem 81:6656–6667.

3. Wang TJ, Larson MG, Vasan RS, Cheng S, Rhee EP, McCabe E, et al. Metabolite profiles and the risk of developing diabetes. Nat Med. 2011;17(4):448–53.

4. Ferrannini E, Natali A, Camastra S, Nannipieri M, Mari A, Adam K-P, et al. Early metabolic markers of the development of dysglycemia and type 2 diabetes and their physiological significance. Diabetes. 2013 May;62(5):1730–7.
